# Parallel detection of theta and respiration-coupled oscillations throughout the mouse brain

**DOI:** 10.1038/s41598-018-24629-z

**Published:** 2018-04-24

**Authors:** Adriano B. L. Tort, Simon Ponsel, Jakob Jessberger, Yevgenij Yanovsky, Jurij Brankačk, Andreas Draguhn

**Affiliations:** 1 Brain Institute, Federal University of Rio Grande do Norte, Natal, RN 59056-450 Brazil; 2 Institute for Physiology and Pathophysiology, Heidelberg University, 69120 Heidelberg, Germany

## Abstract

Slow brain oscillations are usually coherent over long distances and thought to link distributed cell assemblies. In mice, theta (5–10 Hz) stands as one of the most studied slow rhythms. However, mice often breathe at theta frequency, and we recently reported that nasal respiration leads to local field potential (LFP) oscillations that are independent of theta. Namely, we showed respiration-coupled oscillations in the hippocampus, prelimbic cortex, and parietal cortex, suggesting that respiration could impose a global brain rhythm. Here we extend these findings by analyzing LFPs from 15 brain regions recorded simultaneously with respiration during exploration and REM sleep. We find that respiration-coupled oscillations can be detected in parallel with theta in several neocortical regions, from prefrontal to visual areas, and also in subcortical structures such as the thalamus, amygdala and ventral hippocampus. They might have escaped attention in previous studies due to the absence of respiration monitoring, the similarity with theta oscillations, and the highly variable peak frequency. We hypothesize that respiration-coupled oscillations constitute a global brain rhythm suited to entrain distributed networks into a common regime. However, whether their widespread presence reflects local network activity or is due to volume conduction remains to be determined.

## Introduction

Oscillations are ubiquitous in the electrical activity produced by the brain^1^. They can be observed at multiple scales, from spike times of single neurons, through the mesoscopic scale of local field potentials (LFPs), up to more macroscopic EEG and fMRI recordings^2^. Though brain oscillations are usually classified according to their frequency, other factors influence their definition, such as wave shape, recorded region, animal species, and associated behavior^3,4^. These parameters are important because different types of oscillatory activity exhibit a variable range of frequencies that often overlap; moreover, distinct rhythms can also occur within the same frequency range^5,6^. Ideally, oscillations should be classified based on their origin and physiological function. Unfortunately, such a classification is currently not possible since, for most oscillations, we still do not have a full understanding of their underlying mechanisms.

We have recently studied LFP oscillations in rats and mice that fail to conform to traditional frequency-based definitions: the so-called respiration-entrained rhythm (RR)^6–10^. More than 75 years ago, Lord Adrian working with anesthetized hedgehogs had already demonstrated that the brain produces electrical activity phase-locked to breathing cycles^11^; respiration-entrained LFP oscillations have been since well characterized^12–15^. However, such respiration-coupled network oscillations were believed to be mostly restricted to areas involved in olfaction such as the olfactory bulb and piriform cortex^14,16,17^. Recent studies have now revealed that RR can be observed in many more brain areas than previously thought. Namely, RR has been detected in the hippocampus^6,7,9^, parietal cortex^10^, sensory barrel cortex^18^, and prefrontal cortex^6,10,19^. Importantly, since RR follows breathing rate, its peak frequency is quite variable and depends on animal species and behavioral state. In rodents, it often assumes values in the delta and theta frequency range^9,10,15,20^, which may have precluded its identification as an independent rhythm^21^.

In the present work, we sought to expand our previous findings by analyzing LFPs from several brain regions of freely moving mice along with respiration. The recording sites were selected so as to include anatomically distant brain regions, irrespective of their functional roles. To further differentiate RR from theta, we focused our analysis on two behavioral states in which theta oscillations are prominent: exploration and REM sleep. Our results show that respiration-coupled oscillations can be detected in parallel with theta in widespread brain regions, including neocortical areas as well as subcortical structures such as the thalamus, amygdala, and ventral hippocampus. These findings suggest that respiration-coupled network activity could potentially constitute a global brain rhythm, which has not been previously recognized as such due to its variable frequency and the usual lack of simultaneous recordings of respiration in LFP studies. Nevertheless, while we demonstrate that RR can be detected in several brain structures, we also discuss the need for future studies addressing whether the widespread presence of RR is due to volume conduction or local generation.

## Materials and Methods

Local field potentials (LFPs) were recorded from a total of 15 brain regions of freely moving mice, though different subsets of regions were recorded from each individual animal. A total of 57 animals were analyzed during either exploratory behavior or REM sleep (see Table S1 for a list of analyzed regions per animal and behavioral state). In all animals, respiratory activity was recorded simultaneously with LFPs by either using thermocouple sensors chronically implanted into the nasal cavity (exploration) or whole-body plethysmography (REM sleep). Further details are provided below.

### Ethics statement

The present study was carried out in agreement with guidelines of the European Science Foundation^22^, the U.S. National Institutes of Health Guide for the Care and Use of Laboratory Animals^23^, and has been approved by the Governmental Supervisory Panel on Animal Experiments of Baden Württemberg at Karlsruhe (35-9185.81/G-84/13 and 35-9185.81/G-115/14).

### Animal Care and Housing Conditions

C57BL/6 N mice were purchased at 14 weeks of age from Charles River (Sulzfeld, Germany). Animals were housed in groups of four inside a ventilated Scantainer (Scanbur BK A/S Denmark) on an inverted 12/12-h light/dark cycle (light on at 8:00 p.m.) for a minimum of two weeks. Animals had free access to water and food. Following chronic electrode implantation, mice were housed individually. After finishing recordings, animals were killed with an overdose of isoflurane during brain perfusion.

### Animal Preparation

Fifty seven C57BL/6 N mice (34 female and 23 male) were used in the present study. Animals weighed between 21 and 40 g and were from 14 to 40 weeks old. For electrode implantation, animals were anesthetized with isoflurane in medical oxygen (4% isoflurane for induction, 1.5–2.5% for maintenance, flow rate: 1 l per min). For analgesia, 0.1 mg/kg of buprenorphine was injected subcutaneously prior to and 8 h after surgery. Anesthetized animals were mounted on a stereotaxic apparatus (Kopf Instruments, Tujunga, CA) with a custom-made inhalation tube. Body temperature was maintained at 38 °C by a heating pad (ATC-2000, World Precision Instruments). For monitoring the temperature of nasal airflow, two precision fine bare wire temperature sensors (80 µm diameter, Omega Engineering Inc., Stamford, CT; Part No.: 5TC-TT-KI-40-1M) were implanted into the right and left nasal cavity (11 mm anterior, 0.5 mm lateral). After exposure of the skull, holes of 0.5–1.0 mm in diameter were drilled above the following brain structures: dorsal hippocampus (dHIP), ventral hippocampus (vHIP), olfactory bulb (OB), prelimbic cortex (PLC), parietal cortex (PAC), anterior cingulate cortex (ACC), somatosensory cortex (SSC), insular cortex (INS), vibrissal area of the motor cortex (VMC), visual cortex (VC), lateral entorhinal cortex (LEC), central nucleus of amygdala (AMYG), mediodorsal thalamic nucleus (MD), ventroposterior thalamic nucleus (VPL). For stereotaxic coordinates^24^ of electrode positions, see Table 1. Two stainless steel watch screws (1 × 3 mm) over the cerebellum served as ground and reference electrodes. Recording electrodes were made of pairs of varnish-insulated tungsten wires (50 µm, glued together) which were implanted into the depths listed in Table 1, with the exception of three surface locations (OBs, PAC and VC), where epidural recordings were performed using watch screws.

**Table 1:** Stereotaxic coordinates and sample size.

Brain region (Abbreviation)	Coordinates from bregma (mm)			# mice	
	AP	ML	DV	REM	EXP
Olfactory bulb, gran. layer (OBd)	4.50	0.80	1.30	8	8
Olfactory bulb, surface (OBs)	4.50	0.80	dura	8	8
Anterior cingulate cortex (ACC)	1.98	0.35	1.70	8	8
Prelimbic cortex (PLC)	1.54	0.30	2.50	8	8
Vibrissal motor cortex (VMC)	0.98	1.50	0.80	8	8
Somatosensory cortex (SSC)	−1.06	1.40	0.70	8	8
Insular cortex (INS)	−1.06	3.70	3.50	8	8
Amygdala, central nucl. (AMYG)	−1.06	2.20	4.70	7	7
Medio-dorsal thalamus (MD)	−1.46	0.50	3.00	8	8
Ventral posterior thalamus (VPL)	−1.70	1.80	3.70	8	8
Dorsal hippocampus, CA1 (dHIP)	−2.06	1.50	1.50	8	8
Parietal cortex (PAC)	−2.06	1.50	dura	8	8
Visual cortex (VC)	−2.92	2.00	dura	7	7
Ventral hippocampus, CA1 (vHIP)	−3.16	3.00	3.70	8	8
Lateral entorhinal cortex (LEC)	−4.00	3.50	4.50	8	8

AP: anterior-posterior, ML: medio-lateral, DV: dorso-ventral, dura: epidural, EXP: exploration.

### Electrophysiology

Intracranial monopolar recordings began 6 to 7 days after surgery. Animal’s spontaneous behavior in the home cage was assessed by the video tracking system Ethovision XT 9 (Noldus Information Technology, Wageningen, Netherlands). Movements in the home cage or in the whole-body plethysmograph (EMKA Technologies, S.A.S., France, for details see ref.^8^) were detected by 3-D accelerometry. Successive recording sessions of up to 4 h were performed in the animal’s home cage and in the plethysmograph to collect sufficient sections with non-overlapping theta and respiration frequencies (see below). Extracellular signals were filtered (1–500 Hz), amplified (RHA2116 Intan Technologies, LLC), digitized (2.5 kHz) and stored for offline analyses. Intracellular recordings in PAC were performed as described in ref.^10^.

### Data analysis

**Figure 1:**
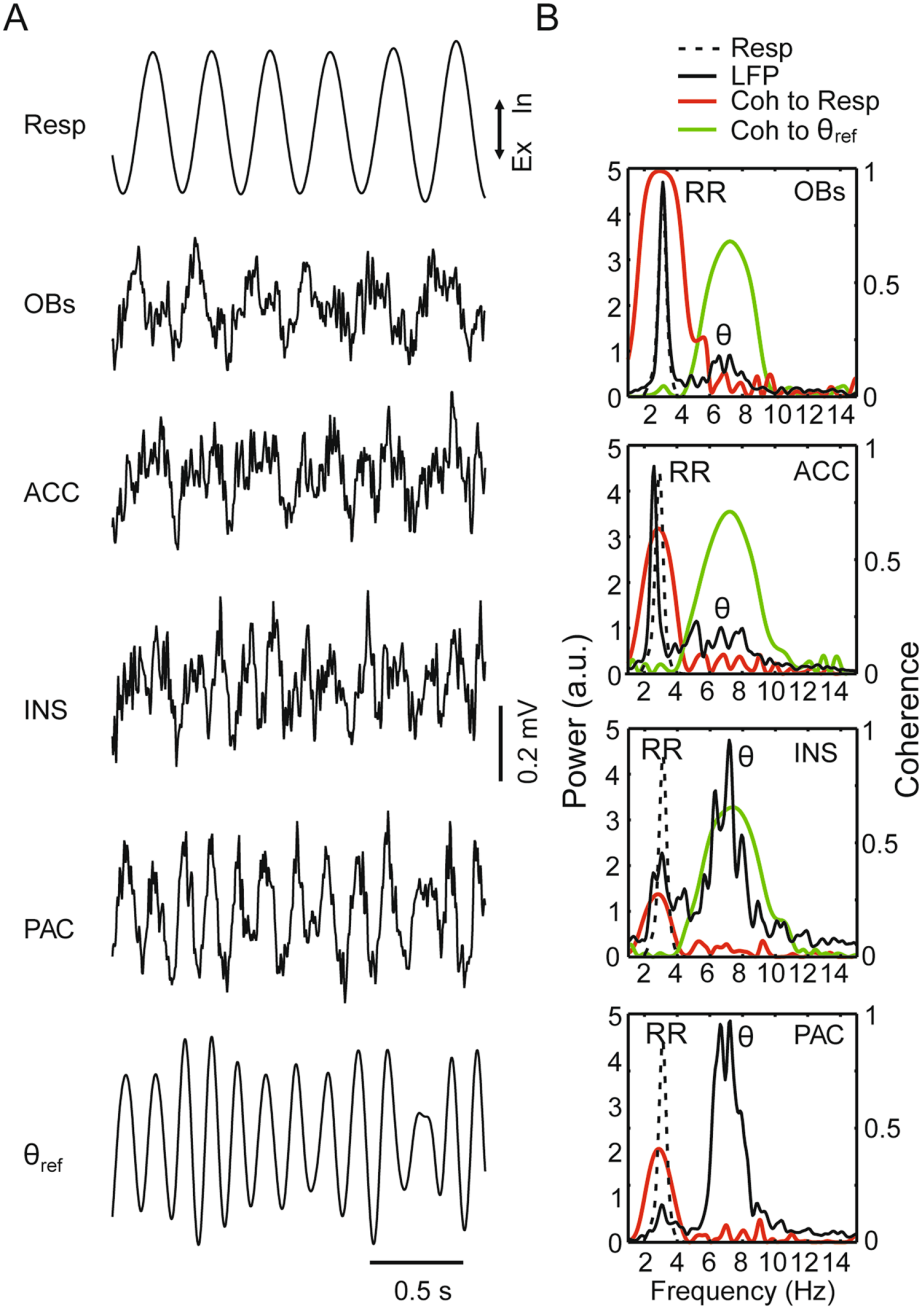
Parallel detection of theta and respiration-entrained rhythms in 4 regions of the mouse brain during REM sleep. (**A**) Traces show two seconds of LFP signals from the olfactory bulb surface (OBs), anterior cingulate cortex (ACC), insular cortex (INS) and parietal cortex (PAC) along with respiration (Resp) and the theta-filtered component of the PAC LFP (θ_ref_). Ex: expiration; In: inspiration. (**B**) Power spectra of LFPs (solid black lines) and Resp (dotted black lines; same in all panels) plotted along with coherence (Coh) spectra between LFP and Resp (red lines) and between LFP and θ_ref_ (green lines). Notice, in addition to theta activity (θ), power and coherence peaks at the same frequency as Resp, which indicates the presence of a respiration-entrained LFP rhythm (RR). Results obtained from simultaneous recordings in a representative animal using 30-s of concatenated REM sleep epochs.

**Figure 2:**
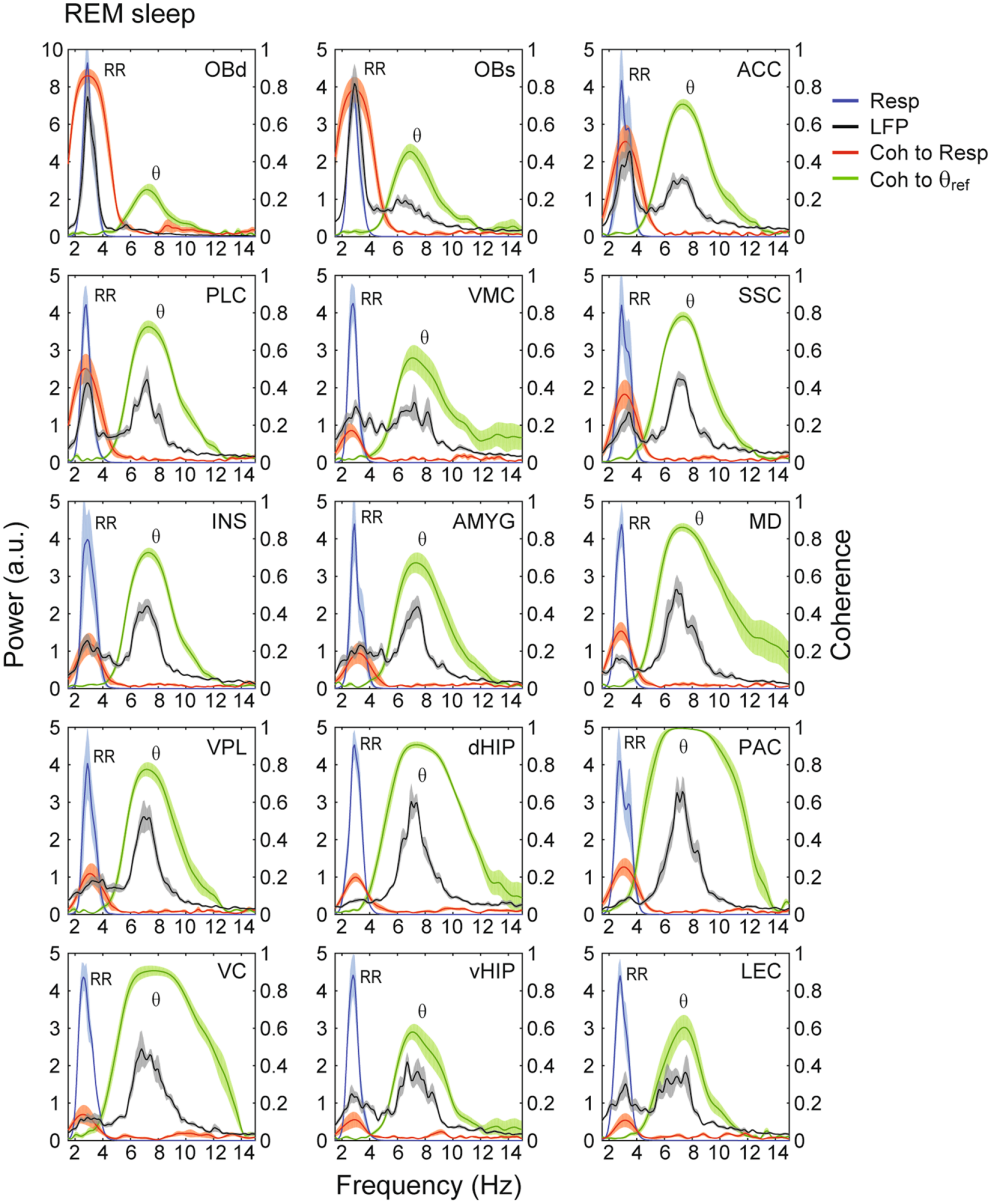
Parallel detection of theta (θ) and respiration-entrained LFP rhythm (RR) throughout the mouse brain during REM sleep. Panels show mean power spectral densities of local field potentials (LFP) (black lines) and their coherence (Coh) to respiration (Resp) (red lines) or theta (θ_ref_) (green lines) in fifteen brain regions during REM sleep (30 seconds of concatenated data per animal). Shades represent ± SEM. The reference theta-filtered signal was taken from either the dorsal hippocampus or the parietal cortex. Respiration was assessed through whole-body plethysmography. Power spectra of respiration are also shown (blue lines). Notice LFP power peaks as well as LFP-Resp coherence peaks at the respiration frequency in all regions. OBd: deep olfactory bulb (granular cell layer); OBs: surface of olfactory bulb; ACC: anterior cingulate cortex; PLC: prelimbic cortex; VMC: vibrissal area of motor cortex; SSC: somatosensory cortex; INS: insular cortex; AMYG: amygdala; MD: mediodorsal thalamus; VPL: ventral posterior lateral thalamus; dHIP: dorsal hippocampus; PAC: parietal cortex; VC: visual cortex; vHIP: ventral hippocampus; LEC: lateral entorhinal cortex.

**Figure 3:**
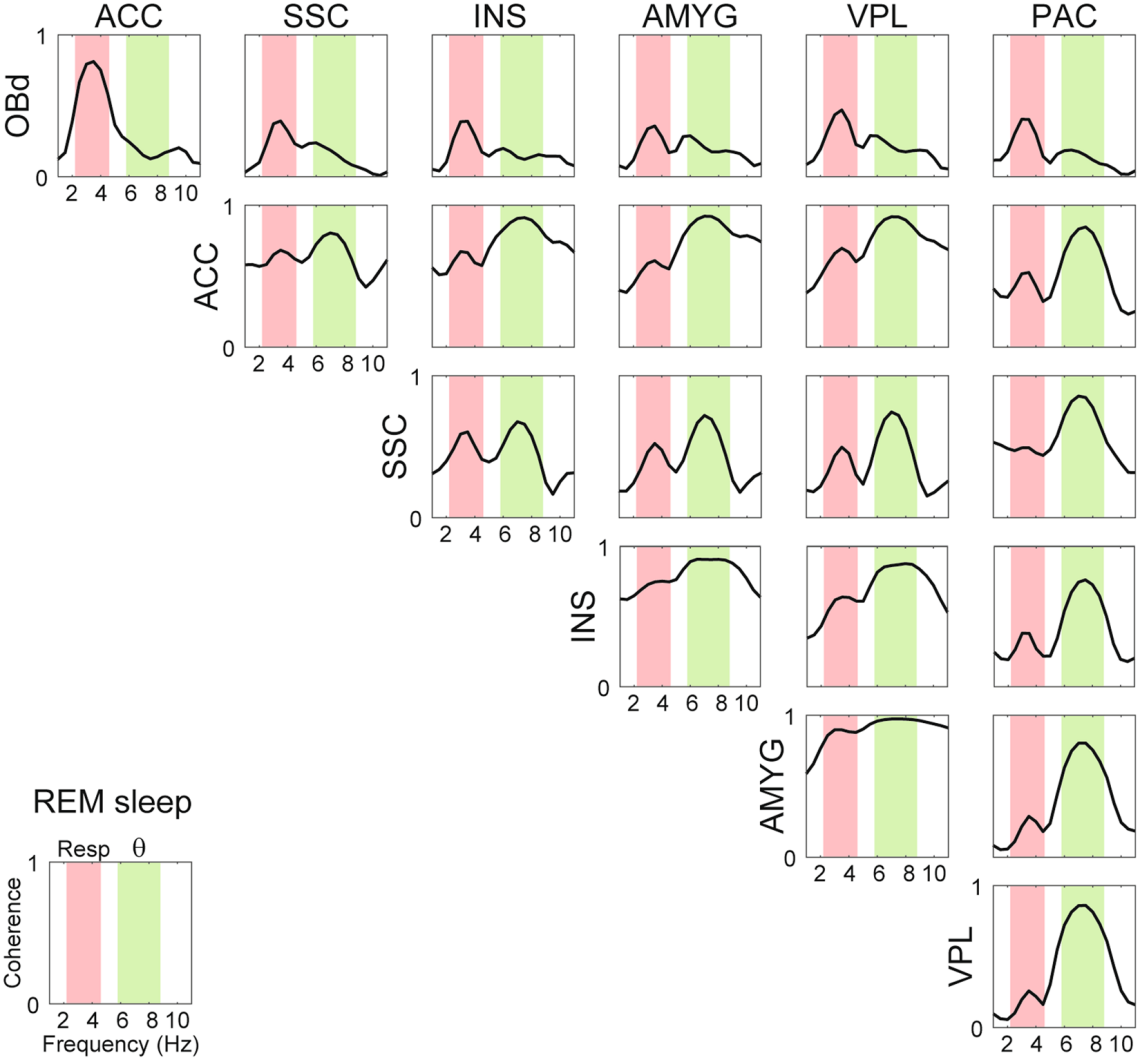
Representative example of cross-regional LFP coherence during REM sleep. Notice coherence peaks at both the respiration and theta frequencies. Similar results were found for all animals, though the exact subset of analyzed regions differed among animals (Table S1).

**Figure 4:**
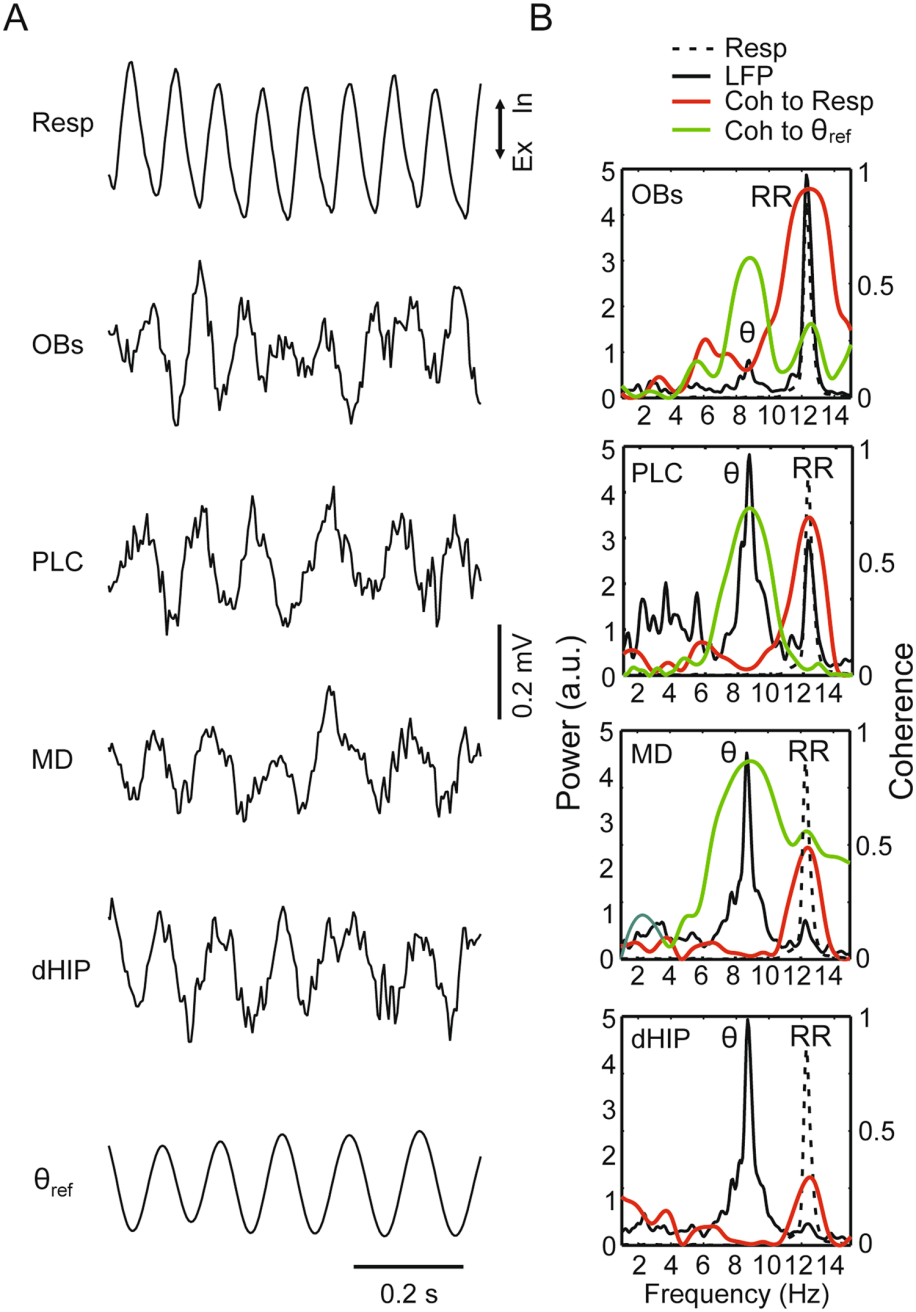
Theta and respiration-entrained rhythms in a representative animal during exploration. (A,B) Panels as in Fig. 1A,B. The reference theta signal (θ_ref_) was obtained from the dorsal hippocampus. In B, we analyzed 30-s of concatenated epochs in which the animal explored the environment while breathing at a rate faster than theta.

**Figure 5:**
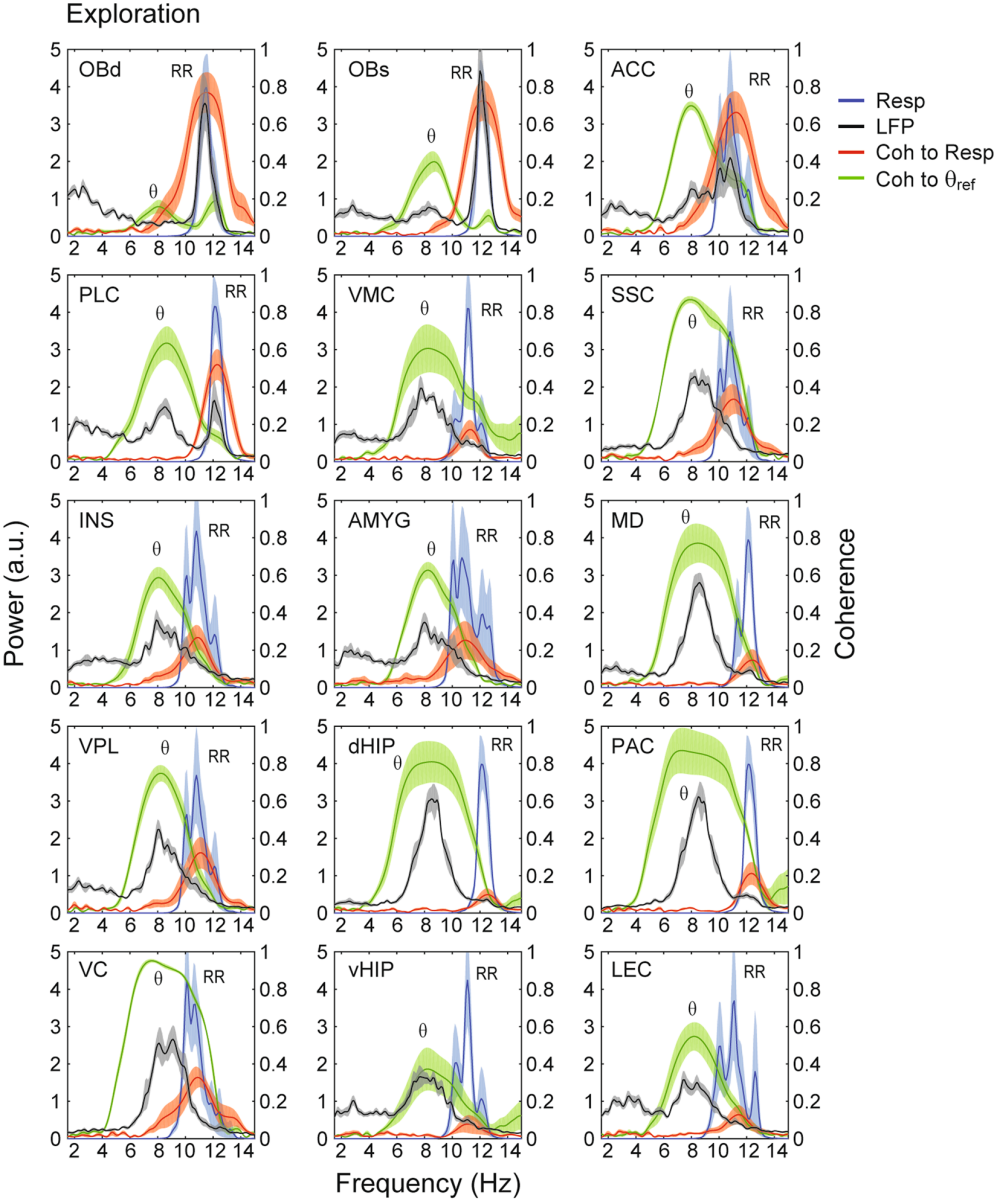
Parallel detection of theta (θ) and respiration-entrained LFP rhythm (RR) throughout the mouse brain during exploration. Panels show the same as in Fig. 2. Each sample consisted of 30 seconds of concatenated data obtained during exploration with respiration faster than theta. The reference theta-filtered signal was taken from either the dorsal hippocampus or the parietal cortex. Respiration was assessed through thermocouples in the nasal cavity.

**Figure 6:**
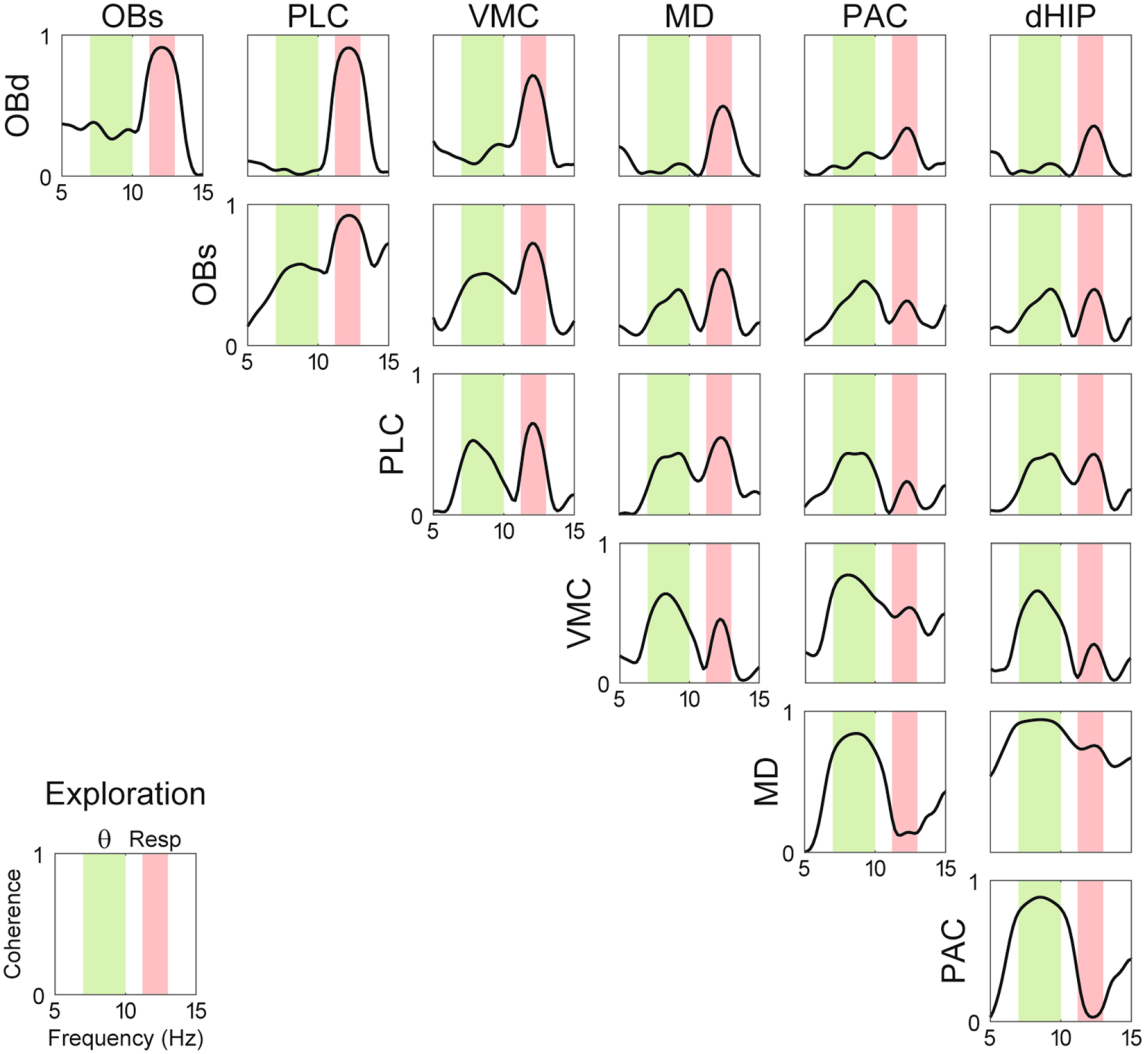
Representative example of cross-regional LFP coherence during exploration. Notice coherence peaks at both the respiratd theta frequencies.

**Figure 7:**
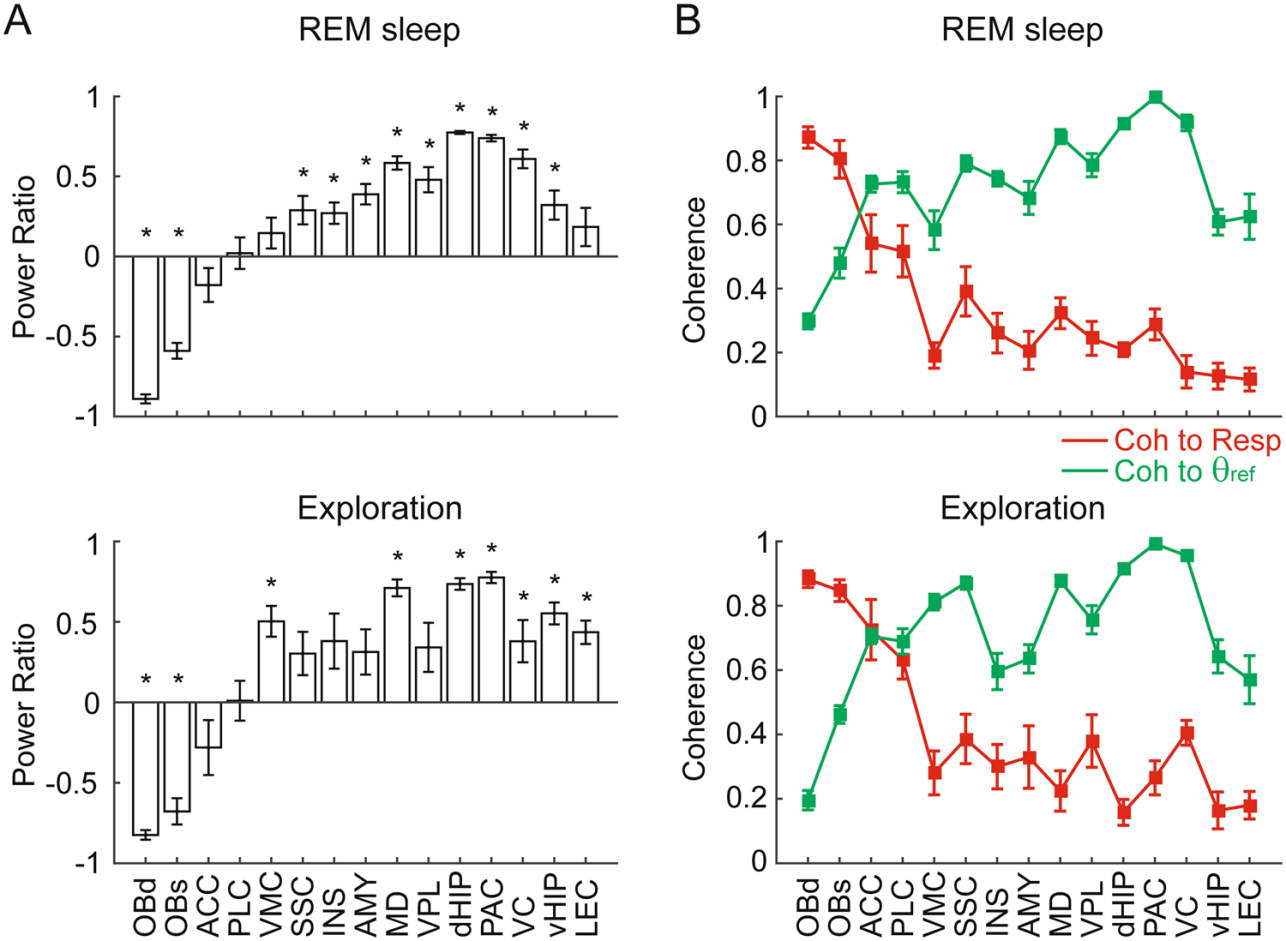
Spatial distribution of the respiration-entrained rhythm (RR) and theta (θ) activity during REM sleep and exploration. (**A**) Mean relative power ratio ( ± SEM) between θ and RR for each recorded region. Note that no brain region exhibits θ alone (power ratio = 1.0) or RR alone (power ratio = −1). *p < 0.05 compared to 0 (paired t-tests). (**B**) Mean LFP coherence to respiration (red) or to theta (green) (±SEM). The reference theta-filtered signal was taken from either the dorsal hippocampus or the parietal cortex. Notice in A and B similar distributions of theta and RR activity during REM sleep and exploration.

**Figure 8:**
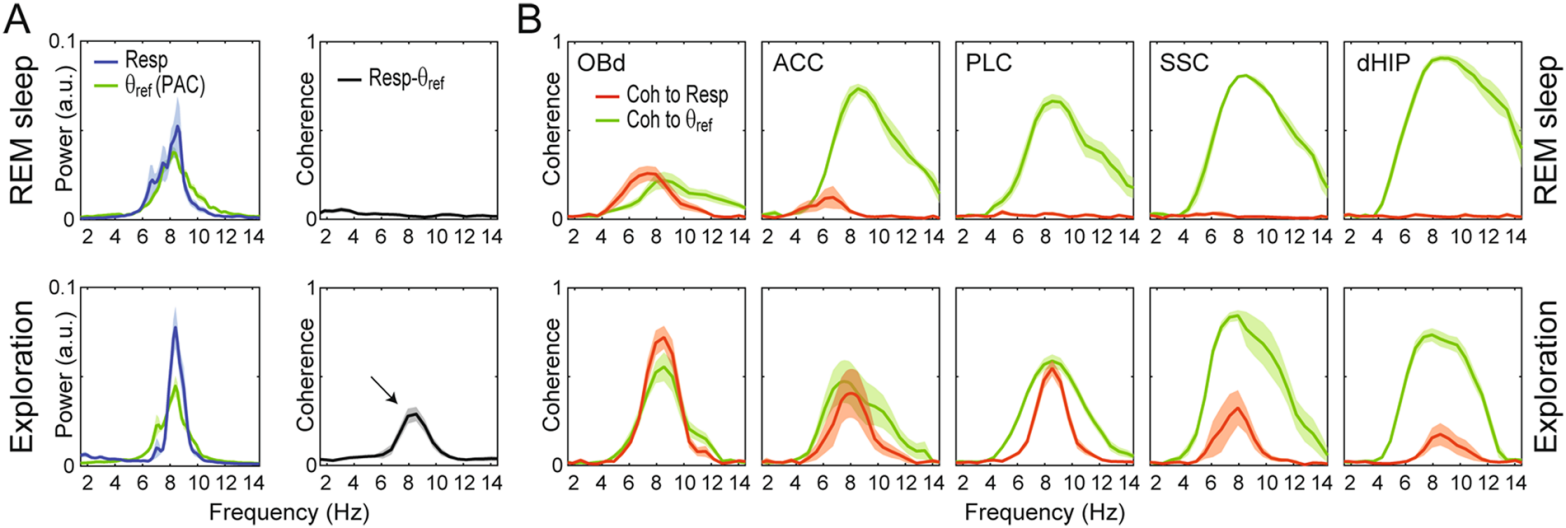
Phase coherence between theta and respiration at overlapping frequencies. (**A**) Power spectra (left) and coherence (right) between respiration and theta (derived from PAC recordings) when both rhythms have the same frequency during REM sleep (top) and exploration (bottom). Notice a coherence peak only during exploration (black arrow). (**B**) LFP coherence to respiration (red) or theta (green).

Data were analyzed in MATLAB (The Mathworks Inc., Natick, MA) using built-in and custom-written routines. We focused on periods of exploration and REM sleep recorded in the home cage and plethysmograph, respectively^8,10,25^. In both states, theta oscillations and respiration may overlap in frequency (see Fig. S1 for the distributions of their instantaneous frequencies during REM sleep and exploration). In Figs 1–7, we only used epochs where respiration frequency (based on the power spectrum of the respiration signal) and theta frequency (inferred by the PAC or dHIP LFP power spectrum) were not overlapping. For each animal and region, the analyzed LFP length was fixed at 30 s, obtained by concatenating epochs within periods of exploration and REM sleep with the largest frequency difference between theta and respiration. In Fig. 8 we selected REM sleep and exploration epochs in which theta and respiration overlapped in frequency.

#### Spectral and coherence analysis

Power spectral density was calculated by means of the Welch periodogram method using 4-s Hamming windows with 50% overlapping (*pwelch.m* function from the Signal Processing Toolbox). To compute LFP phase coherence to either the respiration or the reference theta signal, we used 1-s windows with 50% overlap (*mscohere.m* function from the Signal Processing Toolbox). Filtering into the theta band (5–10 Hz) was obtained by using the *eegfilt.m* function from the EEGLAB toolbox^26^; to remove artifacts, the respiration signal was band-pass filtered using cutoff frequencies around its peak in the power spectrum. The reference theta signal was taken as the theta-filtered LFP from either PAC (preferentially) or dHIP (animals with no PAC recordings). Notice that since dHIP and PAC have very high coherence at theta, similar results are obtained when using either region as the theta reference. The phase-amplitude comodulograms shown in Fig. S4 were computed as previously described^10^.

#### Power ratio

For a fixed region and behavioral state, the relative power ratio was obtained by subtracting the peak power of RR from the peak power of theta, normalized by their sum:

$$Power\,Ratio=\frac{Theta\,power-RR\,power}{Theta\,power+RR\,power}$$

The power ratio varies between −1 and 1; positive values indicate that theta power is stronger than RR power; conversely, negative values indicate that RR power is stronger.

### Histology

After conclusion of the experiments, animals were deeply anesthetized with isoflurane and perfused transcardially with phosphate buffered saline and subsequently with 4% paraformaldehyde (PFA). Brains were carefully dissected, stored in PFA overnight and coronal sections were cut (50 µm), mounted, and stained with cresyl violet. Electrode position was then verified by light microscopy.

### Statistics

For displaying group results (Figs 2 and 5), power and coherence spectra are expressed as means ± SEM over animals. In Fig. 7, power ratio values were compared against zero using paired t-tests. Relative power ratios between prefrontal (ACC and PLC) and posterior (VC and LEC) cortical regions were compared with unpaired t-tests. Coherence values between LFP and either respiration or the theta reference signal were compared against chance using surrogate-based statistical testing. To that end, surrogate values were obtained by computing coherence spectra between LFPs and reference signals (theta or respiration) from different animals. For each region, the actual distribution of peak values in LFP-respiration or LFP-theta coherence spectra (sample size: # of animals) was compared (unpaired t-test) with the distribution of surrogate coherence values at the corresponding frequency (sample size: [# of animals] X [# of animals −1]).

### Data availability

Data are available from the corresponding authors upon reasonable request.

## Results

We analyzed LFPs recorded along with respiration in a total of 57 freely moving mice (see Table 1 for the exact number of animals per region, and Table S1 for the analyzed regions per each individual animal). We focused our analysis in periods in which theta and respiration were not overlapping in frequency. To that end, during REM sleep we selected epochs in which respiration frequency was lower than theta, and, during exploration, epochs in which animals breathed faster than theta.

Figure 1A shows a representative example of simultaneous recordings of respiration and LFPs from four regions during REM sleep. Respiration was assessed through whole-body plethysmography since nasal thermocouple signals are not fully reliable during sleep^8^. Visual inspection of the traces readily reveals that the parietal cortex LFP exhibited a prominent theta rhythm at ~7 Hz, characteristic of REM sleep. On the other hand, the olfactory bulb and the anterior cingulate cortex displayed a slower LFP rhythm that closely followed nasal respiration at ~3 Hz, which we refer to as the respiration-entrained rhythm (RR). Interestingly, the LFP signal recorded from the insula exhibited both RR and theta activity (compare with the parietal cortex and olfactory bulb LFPs). Figure 1B displays respiratory frequency, LFP power spectra from the respective regions and LFP phase coherence to respiration (red traces) or to a reference theta signal (parietal cortex LFP band-pass filtered at 5–10 Hz; green traces). Notice that all LFPs were coherent with the theta-filtered signal at the theta frequency. At the same time, however, LFPs were also coherent with the respiration signal at the respiration frequency. Therefore, in this example case, all regions exhibited both theta and a slower respiration-coupled rhythm during REM sleep, albeit with different relative magnitudes.

Figure 2 shows that similar results hold at the group level during REM sleep, and extends to all fifteen brain regions analyzed (mean RR frequency: 2.97 ± 0.06 Hz; mean theta frequency: 7.10 ± 0.07 Hz). In general, RR was most prominent in frontal regions such as the olfactory bulb, prelimbic cortex, and anterior cingulate cortex. Nevertheless, RR could also be detected at a smaller magnitude in diverse other areas such as the thalamus, ventral hippocampus, and lateral entorhinal cortex. Similarly, theta oscillations could also be detected in several regions, though with lower amplitude in the frontal regions where RR prevailed. Phase coherence between LFP and either respiration or the theta reference signal was significantly higher than chance in all recorded regions, irrespectively of the magnitude of RR and theta (Fig. S2 and Tables S2 and S3). Of note, coherence peaks at both theta and respiration frequencies could also be observed between LFP pairs recorded from different regions (Fig. 3). Therefore, we conclude that RR and theta may be simultaneously detected in widespread regions of the mouse brain during REM sleep. While they are not mutually exclusive, RR is most noticeable at frontal regions while theta dominates more posteriorly.

We next analyzed awake periods in which the animals actively explored the home cage, a behavior that induces robust theta oscillations^10^. Nasal respiration was tracked using temperature sensors implanted into the nostrils. It should be noted that during locomotion and exploration the breathing rate of mice may be the same or even faster than theta frequency^9,10,27^. As previously argued^7,9,10,21^, it can, therefore, be difficult to disentangle both rhythms. Here we opted to focus on exploration periods in which animals breathed faster than theta, inferred by two clearly separate signals in power spectra. Figure 4A shows example traces of LFPs and respiration in a representative animal during such a period. Notice prominent theta oscillations at ~8.5 Hz in the dorsal hippocampus. On the other hand, the olfactory bulb LFP exhibited faster oscillations at ~12 Hz that were clearly locked to nasal respiration, thus characterizing RR activity. Interestingly, as shown in Fig. 4B, the power spectrum of the LFP recorded from the prelimbic cortex revealed two peaks, one at the same frequency as the hippocampal power peak and corresponding to theta oscillations and the other at the same frequency as respiration and corresponding to RR. Notice back in Fig. 4A that it is very difficult to infer the existence of either rhythm solely by visual inspection of the prelimbic cortex LFP trace. This is because the simultaneous presence of both theta and RR leads to alternating effects of constructive and destructive interferences that give rise to frequency beating (see Fig. 3 in ref.^7^). Finally, although not apparent upon visual inspection (Fig. 4A), the power and coherence spectra reveal that RR was also present in the mediodorsal nucleus of the thalamus and in the dorsal hippocampus, but at a much lower magnitude (Fig. 4B). Therefore, and similarly to the example in Fig. 1, during exploration RR and theta could be simultaneously observed in all recorded regions.

Figure 5 shows group results of LFP power spectra and LFP coherence to theta and respiration for 15 brain regions recorded while animals were engaged in exploration with respiration faster than theta (mean RR frequency: 11.03 ± 0.12 Hz; mean theta frequency: 8.50 ± 0.09 Hz). Notice parallel detection of both rhythms in several regions. Consistently, peak coherence values between LFPs and either reference signal (respiration or theta) were significantly higher than chance in all recorded regions during exploration (Fig. S3 and Tables S4 and S5). Figure 6 shows a representative example of inter-regional coherence during exploration; notice peaks at both theta and respiration frequencies for several LFP pairs.

Finally, Fig. 7 provides group data for the spatial distribution of LFP coherence to respiration or theta, as well as of the relative power between theta and RR within each of the 15 brain regions. In the bar graphs (Fig. 7A), a relative power ratio of 1 denotes exclusive theta activity while −1 denotes exclusive RR activity; a relative power ratio of 0 means that both rhythms had the same magnitude. Notice similar distributions of RR and theta during REM sleep and exploration: in either behavioral state, RR was most prominent in frontal regions while theta prevailed in more posterior regions, with no region exclusively exhibiting only theta or RR. Consistently, in both behavioral states the relative power ratio was statistically significantly different between prefrontal (ACC and PLC) and caudal cortical regions (VC and LEC) (REM sleep: t(29) = 3.86, p = 0.0006; exploration: t(29) = 4.22, p = 0.0002).

In all, our results show that not only theta but also RR can be detected in several regions of the rodent brain.

## Discussion

We have simultaneously tracked nasal respiration along with multisite LFP recordings in freely moving mice during two behavioral states classically associated with theta oscillations. For each region, we computed phase coherence spectra between its LFP and either respiration or a reference theta signal. As in previous work, we inferred the detection of a respiration-locked rhythm if (1) the LFP exhibited a power peak at the same frequency as respiration, and (2) if the LFP coherence with respiration peaked at this frequency. We found that the amplitude of RR was highest in frontal regions, such as the olfactory bulb, prelimbic cortex, and anterior cingulate cortex. Nevertheless, a power peak at the same frequency as respiration and coherent with it was also apparent at more ventral and posterior regions, such as the visual cortex, amygdala, ventral hippocampus and lateral entorhinal cortex. In all, our results provide evidence that respiration-locked network oscillations can be detected in several brain regions, including cortical and subcortical structures, where they may be observed concomitantly with theta oscillations.

Our results are consistent with those of Heck *et al*., who reported that LFP and spiking activity phase-lock to respiration in multiple areas of the neocortex of awake head-fixed mouse^28^. Namely, Heck *et al*. showed that delta-frequency modulations are apparent in LFP averages centered at the end of the expiration cycle, from prefrontal to visual cortices^28^. Our results extend these findings to subcortical structures and theta states in freely moving animals, and further show that respiration can also entrain LFP activity at higher frequencies than delta.

Mice and rats can breathe as slow as 1 Hz during quiet states and as fast as 14–15 Hz during running, exploration and sniffing^15,20,27^. Therefore, the respiration-coupled LFP rhythm escapes a narrow frequency-based definition. Its peak frequency can be the same as that of distinct network oscillations: slow thalamo-cortical oscillations as observed during sleep (~0.3–1 Hz)^29^, delta oscillations (1–5 Hz)^30^, theta oscillations (5–10 Hz)^9^, neocortical mu and alpha rhythms (8–12 Hz)^5^, or even oscillations in the low beta range (12–20 Hz)^31^. In particular, in this work we showed examples of RR at 2–4 Hz during REM sleep, i.e. slower than theta (Figs 1–3), and at 10–14 Hz during exploration, i.e. faster than theta (Figs 4–6).

In Figs 1–7, we intentionally avoided selecting epochs in which breathing rate occurred at theta frequency to more clearly demonstrate the distinction between the two rhythms. But these periods often occur, especially during active behaviors such as exploration (see Fig. S1). In fact, seminal work by Macrides *et al*.^32^ and Kay^13^ have examined coherence between hippocampal and olfactory networks during cognitive tasks that depend on olfaction (odor learning and discrimination). These authors concluded that sniffing and OB LFPs may synchronize with the hippocampal theta rhythm at cognitively-relevant periods of the tasks, which would aid sensorimotor integration. On the other hand, our results suggest that sniffing and respiration-locked rhythms in OB rather synchronize with a distinct oscillatory pattern than theta, which nevertheless may occur at theta frequency depending on breathing rate^9^. To gain insight into this possibility, in Fig. 8 we analyze LFP coherence to respiration and to the reference theta signal when both rhythms have the same frequency. During REM sleep, the reference theta signal was not coherent with respiration and most regions – with the exception of OB – were only coherent with theta, which is to say – by definition – that most regions exhibited only theta but not RR (Fig. 8A). Interestingly, however, during exploration the reference theta signal and respiration exhibited some degree of coherence (Fig. 8B), and the LFPs were coherent with both theta and respiration. The observed coherence between respiration and theta-frequency activity during exploration is therefore consistent with the previous findings by Macrides *et al*.^32^ and Kay^13^. However, from this analysis one cannot conclude whether the septo-hippocampal generated theta oscillations were indeed synchronized with respiration, or else whether theta simultaneously existed with RR at the same frequency. We particularly believe the latter was the case: since RR clearly exists independently of theta when animals breathe slower or faster than theta, we deem plausible that RR also exists independently of theta when both have the same frequency.

It should be noted that previous studies have been referring to RR in the olfactory bulb and piriform cortex as “olfactory theta”^14,15,33^. We do not favor such a nomenclature because (1) it may give the idea of a mechanistic link between RR and hippocampal theta oscillations, while we have shown that the two rhythms are independent^6,7,9^ (see also ref.^34^); and (2) it may mask the fact that olfactory areas may exhibit two peaks within the theta band, one due to the classical theta rhythm and another due to RR^9,10^. Moreover, (3) it may also mask the fact that RR can have peak frequencies much below or much higher than the traditional theta frequency range. Again, given its variability in peak frequency, we consider that a narrow frequency-based definition would not be proper for this rhythm.

Our results show that respiration-coupled oscillations can be globally detected. This raises the possibility that previous research on LFP oscillations may have been “contaminated” by RR, which was not recognized due to the lack of simultaneous recordings of respiration in the experiments^21^. As an example, we note that whether the slow oscillations observed during deep sleep and anesthesia (“up-and-down” transitions)^35^ would couple or not to respiration has been disputed, with evidence for^16^ and against^36^. We have recently solved this debate by showing that there are two oscillations of nearby frequency (0.3–1.5 Hz) during these states, one corresponding to the up-and-down transitions and the other to RR^6^. Given the proximity in peak frequency, some studies likely confounded the latter with the former (see ref.^17^).

Similarly, Ito *et al*. have recently described that “delta” oscillations in the barrel cortex couple to respiration^18^. While our results corroborate the observation of such respiration-coupled oscillations in sensory cortices, we would be less inclined to conclude that all delta-frequency activity in the barrel cortex is due to respiration, such as the delta oscillations that occur during sleep in several neocortical regions of rodents^37^. It should be further noted that whisking and breathing have been reported to synchronize^38–40^, and recent work suggested that, depending on behavioral and cognitive demands, these orofacial rhythms would also phase-lock to the hippocampal theta rhythm^41,42^. As argued above, our results instead suggest that coupled sniffing and whisking activity may rather synchronize with a unique LFP rhythm (RR), which may have similar frequency as – but otherwise differs from – septo-hippocampal generated theta oscillations.

As another example, we also believe that the “slow theta” oscillations in the striatum that have been shown to vary in peak frequency during T-maze traversals and to modulate 80–120 Hz oscillations^43^ do most likely correspond to RR (see refs^10,44^). The required re-classification of some slow network oscillations does not, of course, argue against the importance of previous reports. Indeed, we hypothesize that respiration-coupled oscillations provide a mechanism for binding different networks and neuronal ensembles into a common regime, and thus would fulfill similar behavioral and cognitive functions as other rhythms in the same frequency domain. This may even extend to humans where recent work shows the presence, and cognitive relevance, of LFP activity entrained by respiration in hippocampal and amygdala circuits^45,46^.

Importantly, the current and former results show that respiration-coupled oscillations are particularly prominent in the medial prefrontal cortex (mPFC)^6,10,19^. Since RR also modulates gamma in the mPFC^10,19^, we suspect that the coupling between “theta” and gamma recently reported in this region^47,48^ actually corresponds to RR-gamma coupling. Furthermore, theta-frequency oscillations were previously shown to synchronize activity in the mPFC and in the ventral hippocampus during anxiety^49^. Since our results show that both theta and RR exist in these regions, such experiments warrant being revisited with simultaneous recordings of respiration. Finally, we also suspect that the “4-Hz oscillations” described to link mPFC, ventral tegmental area and hippocampus during working memory^50,51^, and to coordinate mPFC and amygdala networks during fear learning^52,53^, are respiration-coupled oscillations (see also refs^54–57^).

While our work shows that RR can be detected in LFPs from widespread regions of the mouse brain, from prefrontal to visual cortices, it remains to be determined whether RR is generated de novo in each of these regions or else if it is volume conducted from olfactory regions. In this sense, being globally detected does not necessarily means being a global rhythm that reflects the activity of distributed local networks from where the recordings were performed. We note that a similar issue applies to theta oscillations: even though theta has been hypothesized to play functional roles in several regions, its local origin in the recorded region has been seldom demonstrated^49,58–62^ (but see ref.^63^). The rostrocaudal gradient reported here, in which RR dominates in frontal regions while theta prevails caudally, is at first glance suggestive of volume conduction from the OB and the hippocampus, respectively. Further suggestive of volume conduction is the fact that several subcortical structures have unstructured network architectures, which would hinder the appearance of mesoscopic LFP oscillations even if they receive oscillatory inputs^64^. These observations highlight some of the limitations in inferring the temporal organization of local network activity solely by LFP analysis, which are not unique to our study^64^.

Local generation of an oscillation is typically inferred by bipolar recordings, current-source density analysis (CSD), or modulation of unit activity in the recorded region. In addition, the modulation of gamma frequency oscillations – which are believed to represent local activity^65,66^ – has also been considered as suggestive of a local effect of slow rhythms^34,66–68^. In this regard, it has recently been shown that RR modulates gamma oscillations in non-olfactory neocortical regions^10,18,19,34^. Respiration-gamma coupling is most widely observed during wake immobility in the absence of theta oscillations^10^. Here we could confirm and extend such findings to more brain regions: we found phase-amplitude coupling between RR and ~70–120 Hz gamma oscillations in most recording sites (Fig. S4). While such a result is suggestive of a local influence of RR, some have argued that volume conduction occurs irrespective of the frequency band^69^. Therefore, it is possible that volume conduction would underlie both the detection of RR and of the modulated gamma activity.

**Figure 9:**
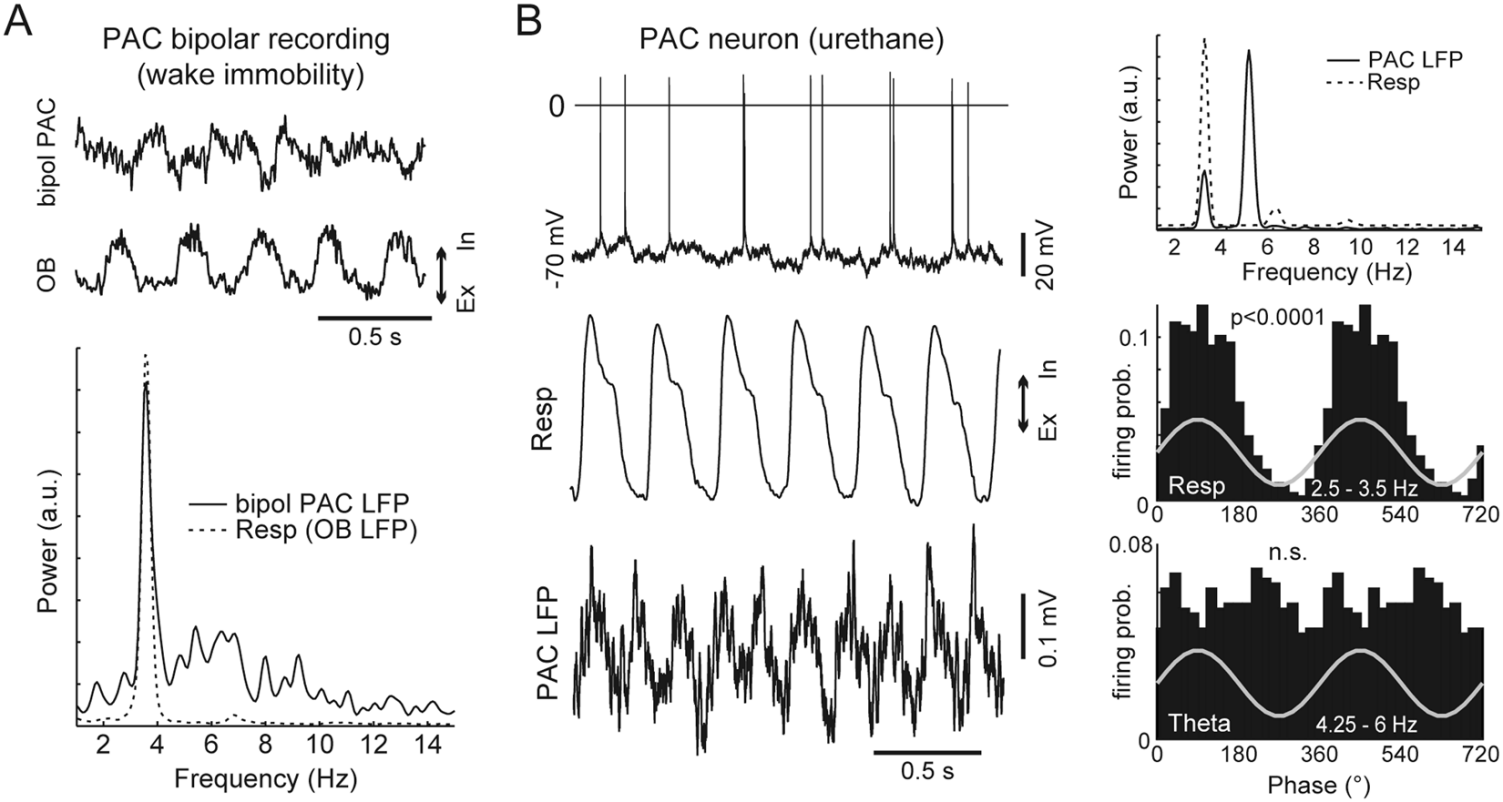
Evidence for the local influence of respiration in the parietal cortex. (**A**) The top trace shows an example LFP recorded during immobility through bipolar electrodes in PAC. The simultaneously recorded OB LFP - used as a proxy of respiration activity in this experiment - is also shown. The bottom panel depicts the corresponding power spectra. Notice a prominent RR power peak in the bipolar recording. (**B**) The left traces show an intracellular recording from a PAC neuron during urethane anesthesia, along with the respiration and LFP signals. The top right panel shows the power spectra. Notice two power peaks for the PAC LFP, which correspond to RR and theta. The bottom panels depict the spike-phase probability for RR and theta. This example neuron was modulated by respiration but not by theta.

Nevertheless, a local influence of RR has been previously established for some brain regions. For instance, laminar analysis has convincingly shown RR to be locally generated in the dentate gyrus of the hippocampus, where it has much higher amplitude than in CA1^6,7,9^. Furthermore, intracellular recordings in anesthetized animals have shown subthreshold membrane potential variations coupled to respiration in hippocampal^7^ and parietal cortex^10^ neurons. RR was also shown to modulate extracellularly recorded spikes in the hippocampus, somatosensory cortex, parietal cortex, and prefrontal cortex, thus suggestive of a local influence in these regions^7,9,10,18,19,70^. In Fig. 9 we show an example of RR detection by bipolar electrodes in the parietal cortex (−2 mm from bregma) during immobility, and of its modulation of intracellularly recorded spike probability during anesthesia. Thus, it is fair to say that there is evidence that RR does impact local networks in some non-olfactory regions. However, despite these well-established cases, for several other regions the local origin of RR (or volume conduction) has yet to be experimentally determined.

In summary, we have shown that respiration-coupled oscillations can be detected in several brain regions, and, based on such a finding, we hypothesize that they constitute a global brain rhythm. The widespread presence of RR was probably not recognized previously due to the usual lack of simultaneous recordings of respiration along with LFPs. We believe that previous functions attributed to oscillations such as slow oscillations, delta, “4-Hz oscillations” and theta could potentially be due to RR activity. In order to test our hypothesis of a global rhythm, future studies should investigate whether the widespread presence of RR is due to volume conduction or local generation.

## Electronic supplementary material

Supplementary information
