# Supplementary material for: Parallel detection of theta and respiration-coupled oscillations throughout the mouse brain
Source: Sci Rep. 2018 Apr 24;8:6432. doi: 10.1038/s41598-018-24629-z (PMC5915406; doi:10.1038/s41598-018-24629-z)
Supplement: Supplementary file 1 — Supplementary information [file 41598_2018_24629_MOESM1_ESM.pdf]

Parallel detection of theta and respiration-coupled oscillations throughout the mouse brain

Adriano BL Tort[1], Simon Ponsel[2], Jakob Jessberger[2], Yevgenij Yanovsky[2], Jurij Brankačk[2], Andreas Draguhn[2]

[1]Brain Institute, Federal University of Rio Grande do Norte, Natal, RN 59056-450, Brazil; [2]Institute for Physiology and Pathophysiology, Heidelberg University, 69120 Heidelberg, Germany.

Supporting Information

4 Supporting Figures + Legends

5 Supporting Tables

Abbreviations: ACC: anterior cingulate cortex; AMYG: amygdala; Coh: coherence; dHIP: dorsal hippocampus; INS: insular cortex; LEC: lateral entorhinal cortex; LFP: local field potential; MD: mediodorsal thalamus; OBd: deep olfactory bulb; OBs: surface of olfactory bulb; PAC: parietal cortex; PLC: prelimbic cortex; Resp: respiration; RR: respiration-entrained rhythm; SSC: somatosensory cortex; Surrog: surrogate; θ_{ref}: theta reference signal; VC: visual cortex; vHIP: ventral hippocampus; VMC: vibrissal area of motor cortex; VPL: ventral posterior lateral thalamus.

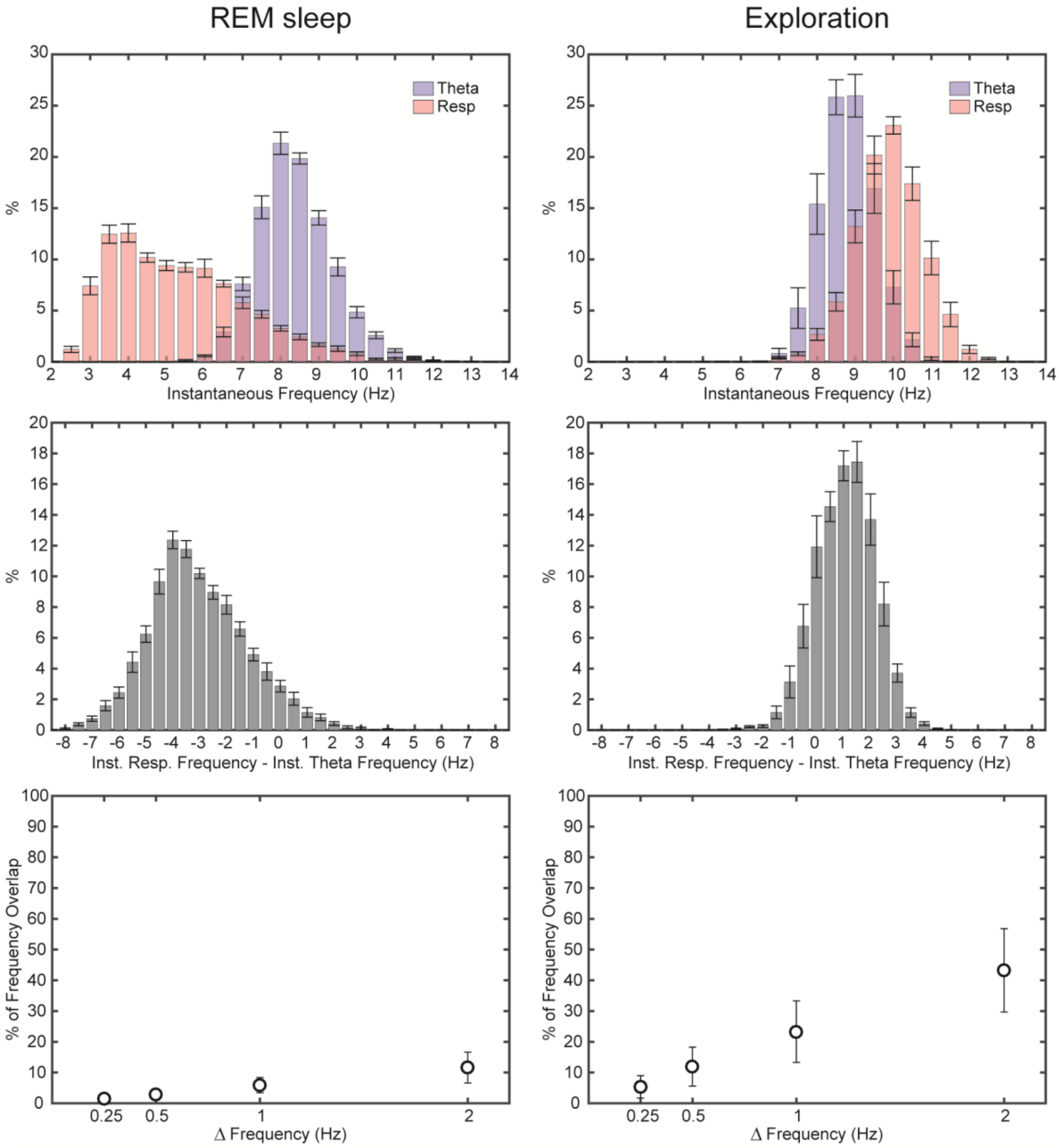

Figure S1. Theta and respiration may overlap in frequency. (Top) Distributions of the instantaneous frequencies of theta (blue) and respiration (red) during REM sleep (left) and exploration (right), computed using non-overlapping windows of 1 second (mean ± SEM over 10 mice; bin width = 0.5 Hz). (Middle) Distribution of the instantaneous frequency difference between theta and respiration (mean ± SEM over 10 mice; bin width = 0.5 Hz). Negative values mean respiration slower than theta. (Bottom) Percentage of frequency overlap as a function of the maximum allowed frequency difference between theta and respiration (mean ± SD over 10 mice).

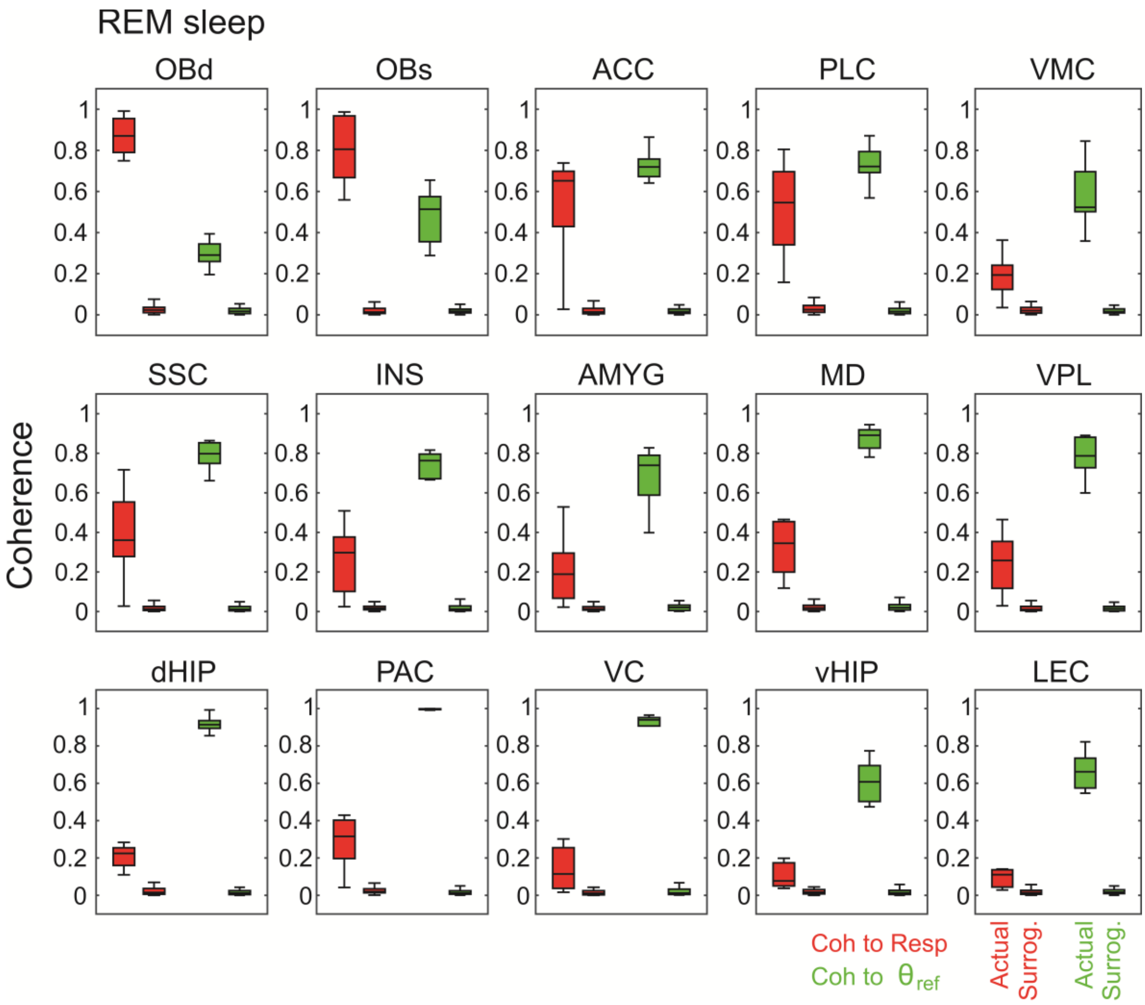

Figure S2. The levels of LFP coherence to either respiration or theta is greater than chance in all recorded regions during REM sleep. Panels show boxplot distributions of the peak coherence values for actual and surrogated data. For each region, surrogated values were obtained by computing phase coherence between the LFP recorded from one animal with the reference signal (respiration or theta) recorded from another animal (see Materials and Methods). This surrogate procedure was performed for all possible pairwise combinations (e.g., for a region with 8 mice recorded, the number of surrogate samples is 8x7=56). The actual and surrogate distributions statistically differ in all recorded regions for both LFP-respiration (red) and LFP-theta coherence (green). See Tables S2 and S3 for statistical analysis.

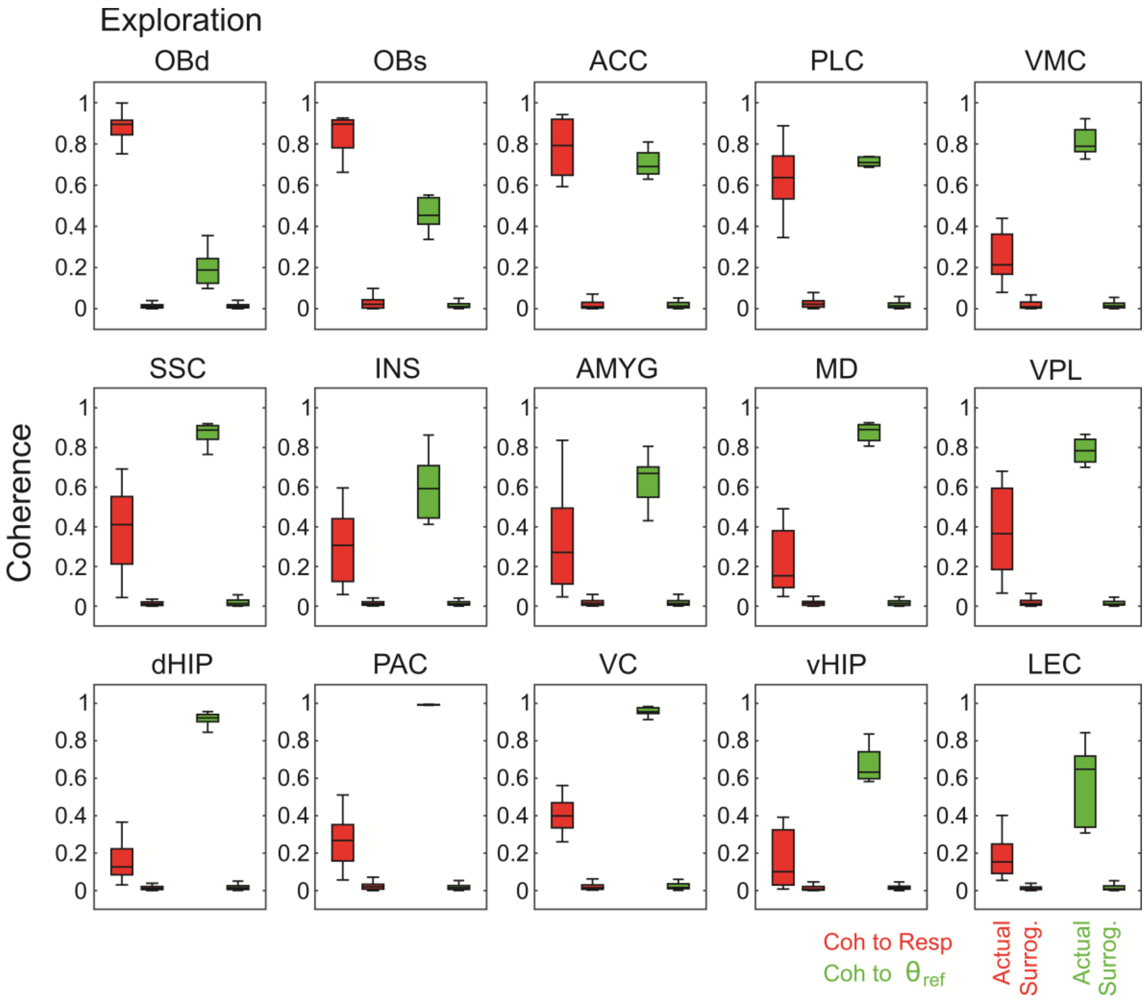

Figure S3. The levels of LFP coherence to either respiration or theta is greater than chance in all recorded regions during exploration. Panels show the same as in Figure S2, but for exploration. See Tables S4 and S5 for statistical analysis.

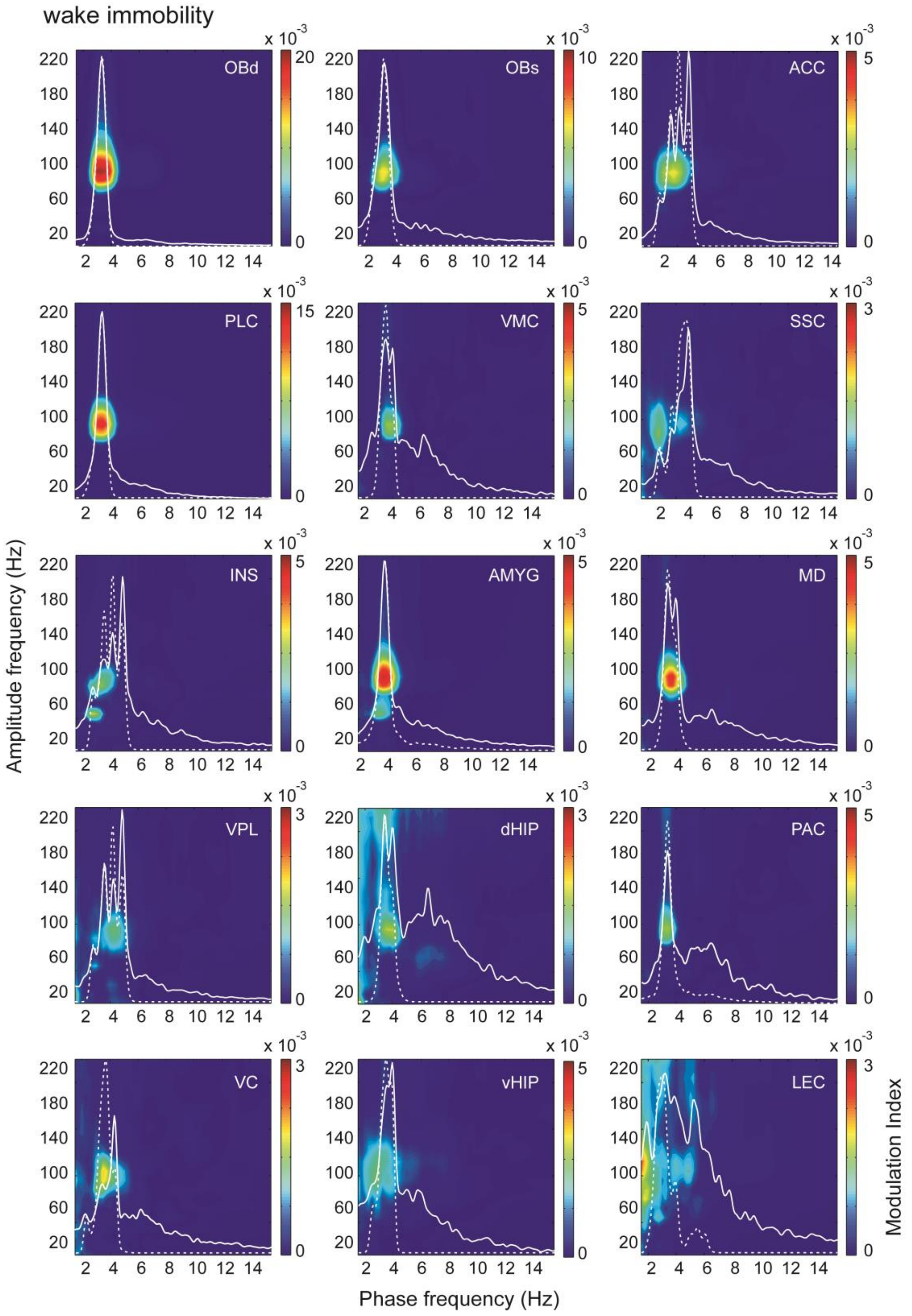

Figure S4. Phase-amplitude comodulograms computed during wake immobility for the 15 recorded regions; warm colors denote cross-frequency coupling (see ref. 10). For each region, the continuous and dashed white lines show the power spectrum of the LFP and respiration signal, respectively (plotted in an arbitrary y-axis scale). The power spectra and comodulograms are group averages. Notice modulation of ~70-120 Hz gamma oscillations by respiration in several brain regions.

Table S1: Analyzed regions per animal and behavioral state

Animal #	REM sleep	Exploration
1		VC
2	dHIP, OBs, VC	VC
3	VC	VC
4	VC	VC
5	VC	dHIP, PAC, PLC, VC
6	dHIP, VC	
7	VC	VC
8	LEC, PAC	LEC, OBs
9	LEC, OBs	LEC
10		LEC
11	dHIP, LEC	LEC
12	LEC, OBs	LEC
13	dHIP, LEC	LEC
14	dHIP, LEC	LEC
15	LEC	dHIP, LEC, OBs, PAC, PLC
16		VC
17	LEC, VMC	
18	MD, PLC, VMC	MD
19		vHIP
20		vHIP, VMC
21	PLC, VMC	OBd, VMC
22	MD, vHIP, VMC	dHIP, MD, OBd, OBs, PAC, PLC, vHIP, VMC
23	MD, vHIP, VMC, OBs	OBd, vHIP, VMC
24	MD, dHIP, VMC, OBs	vHIP, VMC
25		vHIP, VMC
26	OBd, OBs, PAC, vHIP, VMC	vHIP, VMC
27	MD, OBd, vHIP, VMC	MD, OBd, vHIP, VMC
28	AMYG	
29	ACC, AMYG, OBd, SSC, VPL	ACC, AMYG, INS, VPL
30		AMYG, SSC
31	ACC, INS, SSC	
32		ACC, INS, SSC, VPL
33	ACC, INS, SSC, PLC, VPL	ACC, INS, SSC, VPL
34	ACC, PAC, SSC	ACC, AMYG, INS, SSC, VPL
35	ACC, AMYG, INS, OBd, VPL	ACC, AMYG, INS, OBd, SSC, VPL
36	ACC, AMYG, INS, SSC, VPL	ACC, AMYG, INS, SSC, VPL
37	INS, VPL	
38	AMYG, INS, OBd, SSC, VPL	
39	ACC, AMYG, INS, SSC, VPL	ACC, AMYG, INS, SSC, VPL
40	ACC, AMYG, INS, OBd, PAC, SSC, VPL	ACC, AMYG, INS, SSC, VPL
41	MD	MD, dHIP, OBs, PAC, PLC
42	MD, OBs	MD, dHIP, OBs, PAC, PLC
43	PLC	MD, dHIP, OBs, PAC, PLC
44	dHIP, PLC	MD, dHIP, OBs, PAC, PLC
45	MD, dHIP, PAC, PLC	MD, dHIP, OBs, PAC, PLC
46	OBd, PLC	
47	VC	
48	PAC	
49	vHIP	
50	PAC	
51	vHIP	
52	vHIP	
53	PAC, vHIP	
54		OBd
55		OBd
56	PLC	
57	OBd, OBs	OBd

Table S2: LFP-Resp coherence during REM sleep

Region	Mean Coherence		99% Confidence Interval of ΔCoh	*P*
	Actual	Surrogate		
**OBd**	0.871	0.034	0.793-0.882	<0.0001
**OBs**	0.803	0.019	0.726-0.842	<0.0001
**ACC**	0.541	0.021	0.432-0.607	<0.0001
**PLC**	0.516	0.034	0.400-0.564	<0.0001
**VMC**	0.191	0.026	0.115-0.214	<0.0001
**SSC**	0.391	0.018	0.297-0.448	<0.0001
**INS**	0.261	0.021	0.179-0.301	<0.0001
**AMYG**	0.207	0.020	0.128-0.246	<0.0001
**MD**	0.323	0.026	0.244-0.349	<0.0001
**VPL**	0.244	0.016	0.175-0.280	<0.0001
**dHIP**	0.209	0.022	0.159-0.214	<0.0001
**PAC**	0.288	0.028	0.208-0.311	<0.0001
**VC**	0.140	0.020	0.056-0.184	<0.0001
**vHIP**	0.126	0.020	0.065-0.148	<0.0001
**LEC**	0.116	0.017	0.058-0.139	<0.0001

Table S3: LFP-Theta coherence during REM sleep

Region	Mean Coherence		99% Confidence Interval of ΔCoh	*P*
	Actual	Surrogate		
**OBd**	0.297	0.022	0.246-0.305	<0.0001
**OBs**	0.479	0.020	0.413-0.506	<0.0001
**ACC**	0.726	0.022	0.671-0.736	<0.0001
**PLC**	0.732	0.022	0.672-0.747	<0.0001
**VMC**	0.582	0.024	0.491-0.626	<0.0001
**SSC**	0.791	0.019	0.741-0.803	<0.0001
**INS**	0.743	0.022	0.689-0.753	<0.0001
**AMYG**	0.683	0.021	0.610-0.714	<0.0001
**MD**	0.875	0.023	0.824-0.879	<0.0001
**VPL**	0.785	0.022	0.719-0.807	<0.0001
**dHIP**	0.917	0.016	0.882-0.920	<0.0001
**PAC**	0.996	0.017	0.966-0.993	<0.0001
**VC**	0.917	0.023	0.857-0.931	<0.0001
**vHIP**	0.607	0.020	0.543-0.631	<0.0001
**LEC**	0.624	0.021	0.528-0.678	<0.0001

Table S4: LFP-Resp coherence during Exploration

Region	Mean Coherence		99% Confidence	*P*
	Actual	Surrogate	Interval of ΔCoh	
**OBd**	0.882	0.017	0.835-0.896	<0.0001
**OBs**	0.847	0.028	0.778-0.860	<0.0001
**ACC**	0.726	0.019	0.615-0.798	<0.0001
**PLC**	0.632	0.029	0.541-0.666	<0.0001
**VMC**	0.280	0.019	0.194-0.330	<0.0001
**SSC**	0.386	0.015	0.296-0.446	<0.0001
**INS**	0.300	0.017	0.215-0.350	<0.0001
**AMYG**	0.330	0.019	0.216-0.404	<0.0001
**MD**	0.224	0.017	0.145-0.269	<0.0001
**VPL**	0.379	0.020	0.279-0.440	<0.0001
**dHIP**	0.158	0.016	0.101-0.183	<0.0001
**PAC**	0.266	0.027	0.182-0.295	<0.0001
**VC**	0.405	0.023	0.335-0.430	<0.0001
**vHIP**	0.164	0.014	0.092-0.207	<0.0001
**LEC**	0.180	0.014	0.124-0.209	<0.0001

Table S5: LFP-Theta coherence during Exploration

Region	Mean Coherence		99% Confidence	*P*
	Actual	Surrogate	Interval of ΔCoh	
**OBd**	0.196	0.015	0.149-0.212	<0.0001
**OBs**	0.462	0.018	0.412-0.476	<0.0001
**ACC**	0.705	0.019	0.657-0.715	<0.0001
**PLC**	0.689	0.020	0.629-0.711	<0.0001
**VMC**	0.811	0.018	0.764-0.821	<0.0001
**SSC**	0.871	0.019	0.824-0.878	<0.0001
**INS**	0.596	0.017	0.522-0.635	<0.0001
**AMYG**	0.635	0.020	0.568-0.661	<0.0001
**MD**	0.877	0.023	0.822-0.886	<0.0001
**VPL**	0.756	0.018	0.693-0.784	<0.0001
**dHIP**	0.917	0.018	0.879-0.917	<0.0001
**PAC**	0.992	0.017	0.964-0.987	<0.0001
**VC**	0.956	0.024	0.909-0.954	<0.0001
**vHIP**	0.643	0.018	0.574-0.676	<0.0001
**LEC**	0.570	0.019	0.477-0.626	<0.0001
